# Why Maximizing Quality-Adjusted Life Years, rather than Reducing HIV Incidence, Must Remain Our Objective in Addressing the HIV/AIDS Epidemic

**DOI:** 10.1177/2325958218821962

**Published:** 2019-01-22

**Authors:** Bohdan Nosyk, Jeong Eun Min, Xiao Zang, Daniel J. Feaster, Lisa Metsch, Brandon D. L. Marshall, Carlos Del Rio, Reuben Granich, Bruce R. Schackman, Julio S. G. Montaner

**Affiliations:** 1 BC Centre for Excellence in HIV/AIDS, Vancouver, British Columbia, Canada; 2 Faculty of Health Sciences, Simon Fraser University, Burnaby, British Columbia, Canada; 3 Department of Epidemiology and Public Health, Center for Family Studies, Leonard M. Miller School of Medicine, University of Miami, Miami, FL, USA; 4 Department of Sociomedical Sciences, Mailman School of Public Health, Columbia University, New York, NY, USA; 5 Department of Epidemiology, Brown School of Public Health, Providence, RI, USA; 6 Hubert Department of Global Health, Emory Center for AIDS Research, Rollins School of Public Health of Emory University, Atlanta, GA, USA; 7 Independent Public Health Consultant, Washington, DC, USA; 8 Weill Cornell Medical College, New York, NY, USA; 9 Division of AIDS, Faculty of Medicine, University of British Columbia, Vancouver, British Columbia, Canada

**Keywords:** HIV, economic evaluation, economic model, quality-adjusted life years

## Abstract

With efficacious behavioral, biomedical, and structural interventions available, combination implementation strategies are being implemented to combat HIV/AIDS across settings internationally. However, priority statements from national and international bodies make it unclear whether the objective should be the reduction in HIV incidence or the maximization of health, most commonly measured with quality-adjusted life years (QALYs). Building off a model-based evaluation of HIV care interventions in British Columbia, Canada, we compare the optimal sets of interventions that would be identified using HIV infections averted, and QALYs as the primary outcome in a cost-effectiveness analysis. We found an explicit focus on averting new infections undervalues the health benefits derived from antiretroviral therapy, resulting in suboptimal and potentially harmful funding recommendations.

**What Do We Already Know about This Topic?**

We searched PubMed for papers published in English between January 2000, and January 2017, using the terms “HIV”, “AIDS” “cost-effectiveness analysis”, “health economic evaluation”, and “combination intervention”. Our searches retrieved a myriad of economic evaluations in HIV/AIDS, with some favouring the use of incidence averted as health outcome while some favouring utility-based effectiveness measure (i.e. quality-adjusted life years (QALYs) gained or disability-adjusted life years (DALYs) averted). Many health economic evaluation guidelines have endorsed the QALY/DALY-based approach, yet none of the retrieved studies have explicitly discussed the impacts and implications of selecting one over the other as the health outcome.

**How Does Your Research Contribute to the Field?**

Using a previously-validated dynamic HIV transmission model, we evaluated all possible combinations of five distinct interventions that were executed in British Columbia, Canada in a cost-effectiveness analysis. This study found using infections averted as the primary outcome resulted in a different set of ‘optimal’ interventions than QALY-based approach.

**What Are Your Research’s Implications toward Theory, Practice, or Policy?**

Our findings suggest that focusing on averting new HIV infections can lead to sub-optimal decisions as a result of ignoring the health benefits accumulated among the HIV infected population, in particular undervaluing the full benefits of antiretroviral therapy (ART) in mitigating disease progression and mortality among this population. We justified the adoption of QALYs as the basis in assessing the relative value of combination interventions to optimize population health, which is in line economic theory and international best practice guidelines in economic evaluation.

## Introduction

Since the discovery of the preventive benefits of antiretroviral treatment and other prevention strategies, a combination intervention implementation strategy has been proposed to reduce the public health burden of HIV/AIDS.^1^ However, establishing an effective HIV response requires making informed decisions about how best to allocate limited public health funding. Dynamic HIV transmission models can synthesize input data on the spatiotemporal course of an HIV epidemic, as well as incorporate data on existing and emerging HIV care interventions. Model output can then provide comprehensive information to inform decisions about how best to allocate available funding on combinations of HIV treatment and prevention interventions to achieve the greatest health benefit. Modeling can often be the only way to obtain credible evidence of the relative value of combination implementation strategies, accounting for both their costs and population health benefits over the long term.^2^

Motivated by the principle of maximizing population health,^3^ the use of quality-adjusted life years (QALYs) is ubiquitous in health economic evaluation. The QALY is a measure that defines health in terms of time spent in health states, thus capturing improvements in both morbidity and mortality; “disability-adjusted life years” and “life years gained” are similar in principle but measure these constructs more coarsely. Assessments of value from QALY-based cost-effectiveness analyses are directly interpretable, allow for direct comparison across diseases, and are consistent with the theoretical basis of health economic evaluation.^4^ In contrast, cost-effectiveness analyses using other health outcomes (eg, infections averted) may be useful for measuring the effects of particular treatments but do not permit comparisons among diseases and conditions. Panels in the United States^5^ and Britain^6^ and at the World Health Organization (WHO)^7^ have deemed QALYs preferable to alternative measures of health improvement. Backed by an underlying equity principle that equates QALYs gained across disease areas at the population level,^4^ QALYs give priority to interventions that offer the most health benefit in terms of measures people care about: more time spent in good health.^8^

An explicit focus on reducing new HIV infections, for example, by the US National HIV/AIDS strategy^9^ and the National Institutes of Health,^10^ has prompted some to consider HIV infections averted in the denominator of the incremental cost-effectiveness ratio (ICER), or otherwise advocate for the combination implementation strategies on the basis of incidence reduction. This is understandable as it represents a “concrete” outcome that is accessible outside of the scientific community. Further, the notion of incidence reduction no doubt stems directly from the discovery of the secondary preventative benefits of ART,^11^ and also the success of pre-exposure prophylaxis (PrEP) as a new method of prevention.^12^ However, orienting policy and practice to meet this objective instead of using a measure of health benefit such as QALYs may result in suboptimal decisions and pose serious ethical challenges.

The use of incident HIV cases averted is sometimes framed as being directly interchangeable with the use of QALYs in economic modeling studies, or presented alongside cost-effectiveness ratios with QALYs in the denominator.^13^ Epidemiological modeling studies often focus explicitly on averting new HIV infections, implicitly aligning with policies guided on this basis.^14–16^ This is problematic for several reasons. Using HIV cases averted provides no opportunities for comparing value relative to interventions in other disease areas, and we argue here, provides a flawed and ill-conceived perspective on absolute value as well. Most importantly, the immediate and sustained reductions in morbidity and mortality among people living with HIV (PLHIV) receiving ART are not captured. This implicitly places greater value on the lives of individuals at risk of contracting HIV/AIDS than those infected.

We demonstrate the health and equity implications of using HIV infections averted, as opposed to QALYs gained, in judging the relative value of HIV treatment and prevention interventions using a case example from British Columbia (BC), Canada.

## Methods

This case study is built off a model-based evaluation of HIV care interventions presented elsewhere.^17^ The model was adapted and extended upon a previously validated dynamic transmission model previously applied to estimate the health benefits and costs of HIV interventions in the United States, BC, and China.^18^ The model partitioned the adult population into compartments on the basis of gender, HIV risk behavior, screening status, and HIV infection status, as well as CD4 count, diagnosis, and treatment status among the infected population, and explicitly simulated disease progression, as a function of CD4 count, and the dynamics of HIV transmission through homosexual, heterosexual, and needle-sharing contacts. The model was populated with comprehensive linked health administrative and registry data^19^ and validated against 15 external targets. In a prior analysis, we evaluated 5 distinct interventions that were part of a combination implementation strategy executed in BC: HIV testing in hospital, emergency departments (EDs), and outpatient clinic settings, as well as ART initiation and ART retention initiatives.^20–22^ We used observed aggregate-level testing rates and individual-level ART initiation and reinitiation rates during the study period to estimate the independent effects of these interventions. A more detailed description of the model, its inputs and the interventions assessed can be found in a separate manuscript.^17^

In this case study, we take this analysis one step further to assess the impact of all possible combinations of the interventions considered (excluding ART retention interventions, shown to be ineffective in our prior analysis^17^), to compare the optimal sets of HIV care interventions that would be identified using: (a) HIV infections averted, and (b) QALYs as the primary outcomes in a cost-effectiveness analysis. We considered a total of 15 combinations of interventions, plotting them according to their incremental cost and benefit, compared to a status-quo scenario with no additional public health investment. We then plotted health production functions^23^ showing the highest valued combinations of strategies for a range of incremental public health investment over the 28-year study period (3-year intervention implementation period + 25-year time horizon). Combinations falling under the production function generated lower health benefits for a given investment level and were thus weakly dominated strategies.^23^ We plotted the health production functions with both HIV infections averted and QALYs in the y-axes to illustrate differences in valuation using these 2 outcome measures. These results were drawn from the same set of analyses, focusing on one outcome measure as opposed to the other. A third-party payer perspective was applied, accounting for all direct medical and program costs, and we presented all costs in 2015$CDN at an annual discount rate of 3% for both costs and QALYs.

According to best practices guidelines, combinations of strategies lying along the health production function were compared to the next-most resource intensive strategy. The ICERs, represented by the slope of the lines along the production function, can be used in combination with the estimated budgetary impact, communicated in the x-axis, to determine how much a given jurisdiction is willing and able to commit to HIV care strategies. The WHO recommends ICERs <1 times gross domestic product (GDP) per capita per QALY gained to be considered “highly cost-effective,” and <3 times GDP per capita per QALY gained to be considered “cost-effective”^24^ (BC GDP per capita: $55 405). In contrast, it has been proposed that the lifetime treatment cost for a person living with HIV/AIDS (an estimated $420 000 in 2015$CDN^25^) should be the “cost-saving” threshold for HIV infections averted. A jurisdiction’s budget constraint, however, may force selection of a strategy below the recommended threshold.

## Results

**Figure 1.**
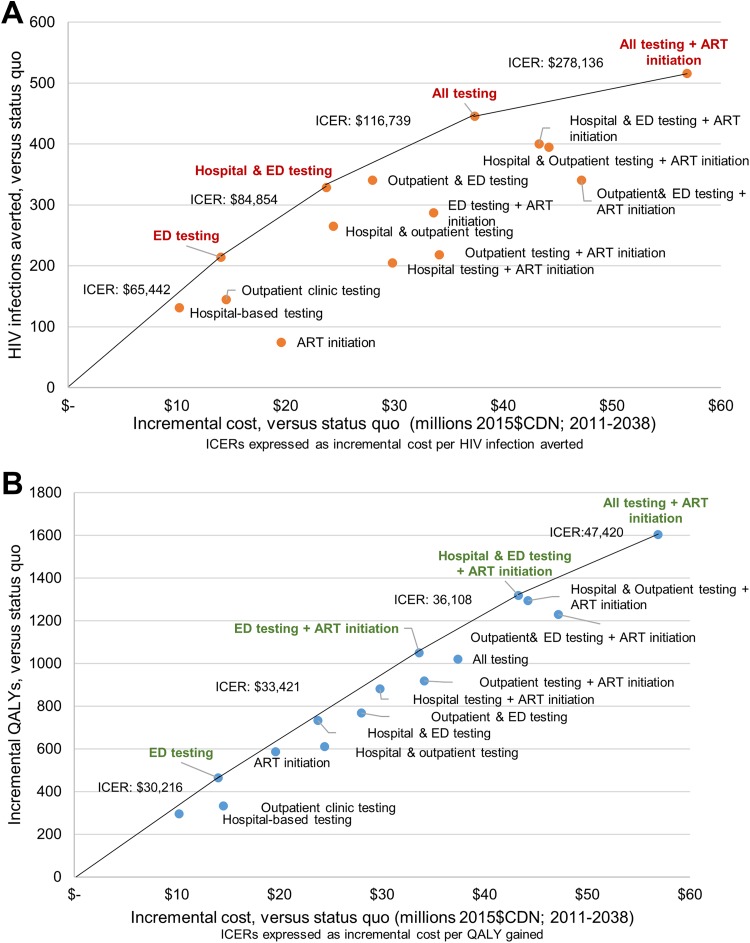
Health production functions to select the optimal combination of HIV care interventions in British Columbia, Canada (2011-2038). **A,** Selection on the basis of infections averted. **B,** Selection on the basis of QALYs gained. QALY indicates quality-adjusted life years.

With HIV infections averted in the y-axis, ED testing, ED + hospital-based testing, all primary care testing and the combined interventions lie on the health production function (Figure 1A). In contrast, with QALYs in the y-axis (Figure 1B), ED testing, ED testing + ART initiation, ED + hospital-based testing + ART initiation, and the combination of all the interventions assessed lie on the health production function. If the funding decision is made without regard for total budgetary impact, the ICERs comparing increasingly resource-intensive strategies along the health production functions indicate the combination strategy would be chosen in both cases, as ICERs compared to less-intensive strategies are below threshold values in both cases. An estimated 516 HIV infections would be averted, including 116 observed in the first 10 years of the study period, 223 observed in the next 10 years, and 177 observed in the final 8 years. However, if the total available funding for the 28-year period is capped at $50 million, a QALY-based approach would identify ED + hospital-based testing + ART initiation as the optimal strategy, while “all primary care testing” would be chosen by attempting to minimize new infections. This decision would result in a net loss of 297 QALYs, borne exclusively by PLHIV.

## Discussion

Using HIV infections averted in the denominator of ICER calculations to assess the relative value of a set of HIV care interventions resulted in a different set of “optimal” interventions identified, as opposed to the evidence-based standard QALY. It should be clear from this exercise that using HIV infections averted in the denominator of the ICER undervalues the full benefits of ART, fully ignoring the direct, individual-level health benefits to PLHIV through delaying or reversing disease progression and thus extending life. We note that while HIV testing in and of itself provides no immediate health benefit to the individual; diagnosis reduces sexual risk behaviours^26^ and provides the basis for subsequent linkage to care, and thus onward transmission. On the other hand, ART initiation immediately slows disease progression and extends life, in addition to its secondary preventive benefits.

Decision-making on the basis of cost-effectiveness analyses estimated with QALYs is not a panacea. Considerable debate rages on the threshold value of the ICER, or the cutoff we should use to determine whether interventions should be considered “cost-effective” or not. A jurisdiction’s “ability to pay,” our selected approach, is one such possibility,^27^ though willingness to pay^28^ and the opportunity cost of displacing existing health services^29^ are other considerations. The debate on the threshold ICER value will no doubt continue; however, the methodology and theoretical underpinnings of the QALY-based approach nonetheless have widespread support in the scientific literature.^8,30^

Although the use of HIV infections averted may hold some intuitive appeal, the relative value of interventions focused on HIV, compared to other disease areas, cannot be compared. Further, while it may be tempting to use the lifetime cost of medical care for PLHIV as a threshold for HIV infections averted, this is not technically correct. Via second- and third-order transmission, incident cases may be averted long after an intervention is initiated, with costs attributable to HIV infection only incurred after diagnosis. Therefore, the majority of these costs may fall outside study time horizons in model-based analyses. Even so, the costs (and benefits) of averted HIV cases are captured explicitly in a dynamic HIV transmission model, so considering an intervention “cost saving” if the ICER is below $420 000/infection averted is patently false. A positive number in the numerator of the ICER necessarily means higher incremental costs for the intervention compared to the status quo.

Whether used in the denominator of an ICER or otherwise set as the focal end point of a modeling study, an explicit focus on reducing HIV incidence is potentially misleading and antithetical to the central principle of health economic evaluation that resource allocation decisions should be made toward optimizing the health of the population. Despite the intuitive appeal and apparent momentum of incidence reduction as the primary objective of public health campaigns to address HIV/AIDS, we argue maximizing QALY gains should form the basis for selecting combination implementation strategies to reduce HIV-related morbidity, mortality, and transmission, and thus maximize population health.
